# Triple-Olfactory Mechanism Synergy: Development of a Long-Lasting DEET–Botanical Composite Repellent Against *Aedes albopictus*

**DOI:** 10.3390/insects17010098

**Published:** 2026-01-14

**Authors:** Chen-Xu Lin, Xin-Yi Huang, Yi-Hai Sun, Bi-Hang Lan, An-Qi Deng, Le-Yan Chen, Qiu-Yun Lin, Xi-Tong Huang, Jun-Long Li, Cheng Wu, Li-Hua Xie

**Affiliations:** 1 Department of Pathogen Biology, School of Basic Medical Sciences, Fujian Medical University, Fuzhou 350122, China13385909928@163.com (B.-H.L.);; 2 School of Life Science, Nanchang University, Nanchang 330031, China

**Keywords:** *Aedes albopictus*, repellent formulation, DEET-citronella-camphor synergy, orthogonal experimental design, mosquito-borne disease control

## Abstract

Mosquito-borne diseases, including dengue fever, chikungunya, and Zika, continue to pose a substantial global public health challenge. This is largely attributable to the absence of effective vaccines and the expanding distribution of vectors such as *Aedes albopictus* (*Ae. albopictus*). Repellents, therefore, remain a critical component of prevention strategies for disease prevention. However, existing formulations have notable limitations. Synthetic repellents such as DEET provide broad-spectrum efficacy but may raise safety concerns, especially at high concentrations. In contrast, botanical repellents, such as citronella and camphor oils, offer more favorable safety profiles but are restricted by short protection durations due to their high volatility. To overcome these drawbacks, this research developed a composite mosquito repellent through the strategic combination of DEET (5–15%), citronella oil (10–20%), and camphor oil (5–15%). This formulation leverages interactions across multiple olfactory pathways to simultaneously enhance efficacy and reduce the DEET concentration. Orthogonal experimental optimization identified an optimized formulation, Mix-3 (consisting of 15% DEET, 15% citronella oil, and 10% camphor oil in 75% ethanol), which achieved a mean complete protection time of 9.45 h. Mix-3 provided longer protection than 7% DEET (mean difference = 5.50 h, *p* < 0.001), 4.5% IR3535 (2.83 h, *p* < 0.001), 10% citronella oil (3.58 h, *p* < 0.001), and 15% DEET (6.50 h, *p* < 0.001). Catnip oil did not contribute significantly to repellency (*p* = 0.895). This study demonstrates that the rational combination of synthetic and botanical repellents effectively overcomes the limitations of single-agent formulations, providing a long-lasting and scalable approach for vector control.

## Simple Summary

Mosquitoes serve as vectors for serious diseases such as dengue fever. However, conventional chemical repellents may contain ingredients that pose risks to human health and the environment. To devise an alternative formulation, this study combined a commonly used chemical repellent with natural plant-derived oils. Mixtures of DEET, citronella oil (derived from lemongrass), camphor oil, and catnip oil were tested to determine the optimal formulation for repelling *Aedes albopictus*. Using scientific blending methodologies, it was found that a blend consisting of 15% DEET, 15% citronella oil, and 10% camphor oil in alcohol demonstrated the greatest repellent efficacy. In laboratory arm-in-cage tests, this mixture provided protection against mosquito bites for 9.45 h, which was significantly longer than that of repellents such as 15% DEET or 10% citronella oil used alone. Notably, this optimized formulation demonstrates enhanced effectiveness through the synergistic combination of DEET with botanical oils. This novel formula presents a hybrid mosquito repellent formulation combining synthetic and botanical actives, demonstrating extended protection time under controlled laboratory conditions. If proven effective and safe in field scenarios, it could assist communities in preventing mosquito-borne diseases.

## 1. Introduction

Mosquitoes pose a major threat to global public health and are recognized as the world’s deadliest animal vector. According to the World Health Organization (WHO), mosquito-borne diseases account for approximately 725,000 deaths each year—a mortality burden far exceeding that caused by predators such as lions, sharks, or snakes. Diseases transmitted by mosquitoes, including dengue fever, malaria, and Japanese encephalitis, continue to impose substantial global public health and socioeconomic impacts. Among competent vectors, *Aedes albopictus* (also known as the Asian tiger mosquito) has emerged as a key transmitter of dengue, chikungunya, and Zika viruses [1,2,3]. Despite extensive vector control efforts, the absence of widely available vaccines for most mosquito-borne diseases necessitates strong reliance on personal protective measures [4]. Therefore, preventing mosquito bites is one of the optimal methods to reduce disease incidence. Accordingly, preventing mosquito bites remains one of the most effective and immediate means of reducing disease transmission.

Repellents constitute a cornerstone of integrated mosquito-borne disease management programs [4]. Current commercial repellents fall broadly into two categories: synthetic and botanical agents. Synthetic repellents—particularly N,N-diethyl-meta-toluamide (DEET)—are the most extensively used. Several other repellent compounds, including picaridin, IR3535, and PMD are also widely applied and exhibit distinct safety and efficacy characteristics [5,6]. DEET remains the benchmark insect repellent, supported by decades of regulatory review and safety assessments; typically found in commercial formulations at concentrations ranging from 10% to 30% for general use, the U.S. Environmental Protection Agency (EPA) permits formulations containing up to 98% DEET following comprehensive evaluations [7]. When used as directed, DEET has a well-characterized safety profile with a low incidence of serious adverse events [8,9]. Nevertheless, interest persists in reducing synthetic repellent load through combinations with botanical co-actives, provided their human health and environmental safety are thoroughly evaluated. Recent findings reporting a potential association between prolonged or frequent DEET exposure and elevated coronary heart disease risk have heightened safety concerns [10], particularly among vulnerable populations such as pregnant women and children, whose physiological susceptibility may amplify risk [11]. This study, therefore, investigates an alternative strategy: combining DEET with botanical oils to evaluate the potential synergistic enhancement of repellent efficacy.

Botanical repellents, including citronella, lemon eucalyptus, and clove essential oils, exhibit documented activity against *Aedes aegypti* and other mosquito species [6]. Plant-based repellents have a long history of traditional use as a personal protection tool against various species of mosquitoes [12]. Their bioactive constituents—terpenoids, alkenes, esters, alcohols, and ketones—are secondary metabolites which evolved as defense mechanisms against herbivores and insects [13]. Essential oils such as citronella, catnip oil and camphor have gained increased attention as lower-toxicity alternatives to purely synthetic repellents [14,15]. However, botanicals are not devoid of toxicological or environmental considerations. For example, catnip acts as an irritant by interacting with the conserved transient receptor potential ankyrin 1 (TRPA1) receptor [16]. Furthermore, essential oil (EO) blends have been increasingly explored for spatial repellents [17]. Despite their favorable safety and environmental profiles, botanical repellents are limited by rapid evaporation and short protection duration, necessitating frequent reapplication and reducing user compliance.

Given these constraints, there is a critical need to develop repellent formulations that balance high efficacy with favorable safety and sustained protection. This study introduces a composite repellent combining DEET, citronella oil, and camphor essential oil, designed to achieve synergistic repellency through complementary mechanisms while mitigating potential side effects. Through a systematic blending approach, this strategy aims to overcome the existing dichotomy between “high-efficacy but high-toxicity” synthetic agents and “low-toxicity but short-duration” botanical agents. Accordingly, *Ae. albopictus* was selected as the target model for this study due to its dominance in the study region and its aggressive expansion into temperate zones where *Ae. aegypti* is less prevalent.

We describe the formulation optimization using orthogonal experimental design and evaluate the repellent efficacy of the resulting mixtures against *Ae. albopictus*. The overarching objective is to establish a scientific foundation for developing repellent solutions that deliver both high efficacy and extended duration, with the long-term aim of supporting environmentally sustainable and scalable public health applications.

## 2. Materials and Methods

### 2.1. Mosquito Collection and Rearing

Two laboratory strains of *Ae. albopictus* were used in this study: the Foshan strain (provided by Professor Chen Xiaoguang’s group at Southern Medical University) and the Fuzhou strain, which was field-collected using ovitraps in Fujian Province from May to October 2024. The Foshan strain is a standard laboratory susceptible strain, while the Fuzhou strain represents a field-collected population with an unknown resistance profile, used here to validate efficacy against wild-type vectors. Both strains were included to assess whether repellent activity remained consistent across genetically distinct populations. Each formulation was tested in more than three replicates using the Foshan strain and more than three using the Fuzhou strain.

Mosquitoes were maintained under controlled insectary conditions (14:10 h light–dark cycle, 26 ± 1 °C, 65 ± 10% RH). Larvae were fed commercial fish food, and adults were provided with a 10% sugar solution. Prior to testing, adult mosquitoes were allowed to mate for 5 days after emergence and had no previous access to blood meals to ensure normal host-seeking behavior.

### 2.2. Compounds Used to Produce Odorant Blends

Following a comprehensive review of mosquito repellents, three EOs and DEET were selected for evaluation (Table 1). These compounds were chosen due to previously reported mosquito-repellent activity [11,12,13]. All test formulations were prepared by dissolving the components in 75% ethanol. Mixtures were homogenized by trituration for one minute to ensure complete dissolution and triturated again immediately before use. Samples were stored at 4 °C in sealed containers to minimize concentration changes caused by volatility.

### 2.3. Bioassays

All experiments were performed following the Chinese national standard GB/T 13917.9-2009 [18]. Twelve healthy adult volunteers (6 males, 6 females, aged 19–35 years; Supplementary Table S1) were enrolled. Each participant tested all 16 formulations from the L_16_(4^3^ × 3^1^) orthogonal design matrix (Table 2) in a randomized within-subject design. Treatment order was assigned using a Latin square arrangement to minimize order effects. Individuals with a history of dermatological disease or allergic reactions to arthropod bites were excluded. All participants were informed of the procedures and potential discomforts before providing written informed consent. And reactions were defined according to established criteria [14].

Bioassays were carried out under ambient conditions of 25–27 °C and 55–75% humidity within a 30 cm × 30 cm × 30 cm cage. Approximately 30 female mosquitoes (3–8 days post-emergence; starved for 6 h) were used per trial. Host-seeking activity was confirmed before each trial by inserting an untreated control forearm into the cage for 2 min; trials proceeded only if ≥10 landings or probings occurred within 30 s [19]. On each volunteer, a 5 cm × 5 cm square was marked on the dorsal hand. A micropipette was used to apply 37.5 μL of the test formulation (1.5 μL/cm^2^), which was evenly spread over the area. To standardize exposure, participants wore custom gloves, leaving only 4 cm × 4 cm of skin area uncovered. Each time after application, volunteers inserted the treated hand into the cage for a 2 min observation period of mosquito landings with probing attempts (defined as tarsal contact with the skin + proboscis extension, in accordance with the WHO 2009 guidelines [19]). Mosquitoes were gently removed immediately upon landing. If a landing with a probing attempt occurred, the repellent was deemed ineffective, and the protection time was recorded. If no probing occurred, the test was repeated hourly up to 3 h, and then every 30 min thereafter until the first confirmed probing attempt. Complete protection time was defined as the interval between application and the first landing with probing attempt. Each formulation was tested with more than six replicates (n = 6 human volunteers, one test per volunteer), including three replicates with the Fuzhou strain and three with the Foshan strain.

The study was conducted in accordance with the Declaration of Helsinki and approved by the Ethical and Biosafety Committee of Fujian Medical University (protocol NIACUC FJMU-0355, approval date 18 November 2024).

### 2.4. Scheme of Orthogonal Design

The orthogonal test design followed a standard workflow consisting of (i) identifying test factors and levels, (ii) selecting an appropriate orthogonal table, (iii) constructing the test scheme, (iv) recording the results, and (v) performing range and regression analyses to determine the optimal factor combination. Four components were evaluated for their contribution to repellent efficacy against *Ae. albopictus*: DEET concentration, citronella oil concentration, catnip oil concentration, and camphor oil concentration. Each factor contained three or four levels (Table 2), denoted by A, B, C, and D.

**Table 2:** Factors and levels used in the orthogonal design.

Level	Factor			
	DEET (A)	Citronella Oil (B)	Catnip Oil (C)	Camphor Oil (D)
1	5%	5%	5%	5%
2	10%	10%	10%	10%
3	15%	15%	15%	15%
4	-	20%	20%	20%

The evaluation criteria included percentage, duration of effectiveness, and degree of skin irritation, each scored on a standardized scale. Duration was measured as the time elapsed until the first mosquito landings with probing attempts, which was recorded in minutes. All formulations were tested in 8–10 replicates under uniform environmental conditions (26 ± 1 °C, 65 ± 10% RH).

The L_16_(4^3^ × 3^1^) orthogonal table was selected to accommodate the required combinations of active ingredient concentrations. Sixteen unique formulations were generated from the factor-level matrix (Table 3).

### 2.5. Comparative Efficacy Testing

To benchmark the optimized formulation, its efficacy was compared with common commercial repellent components, including 7% DEET, 4.5% IR3535, 15% DEET, and 10% citronella oil. A mixed-effects Analysis of Variance (ANOVA) model was used, with subject ID included as a random effect to account for inter-individual variation. Fixed effects included the concentrations of DEET, citronella oil, catnip oil, and camphor oil concentrations. A parallel-group efficacy test was conducted under controlled laboratory conditions. All assays were conducted under controlled laboratory conditions.

### 2.6. Statistical Analysis

In the orthogonal design, one-way ANOVA was applied to identify the most effective odorant blends. Mix-3 was compared with major commercial repellent components using one-way ANOVA followed by Tukey’s HSD test. A *t*-test was used to compare the optimized formulation with individual control treatments. Statistical analyses were performed using SPSS version 20.0 statistical software (IBM, Armonk, NY, USA), and Prism version 8 (GraphPad Software, San Diego, CA, USA) was employed to plot the figures.

## 3. Results

### 3.1. Orthogonal Optimization Identifies DEET–Citronella–Camphor as the Optimal Synergistic Combination

An orthogonal experimental design (L_16_(4^3^ × 3^1^)) was utilized to systematically evaluate and optimize the combinatorial ratios of four repellent compounds. ANOVA results showed that DEET (*F* = 24.495, *p* < 0.001, partial *η*^2^ = 0.36), citronella (*F* = 5.505, *p* = 0.002, partial *η*^2^ = 0.16), and camphor (*F* = 3.187, *p* = 0.028, partial *η*^2^ = 0.102) each had significant effects on protection time. Among these factors, DEET accounted for the largest proportion of variance, aligning with its well-established mechanism of action, whereas citronella and camphor contributed smaller yet statistically significant additive effects. In contrast, catnip oil did not exhibit a significant influence within the tested concentration range (*F* = 1.299, *p* = 0.280) (Table 4).

**Figure 1:**
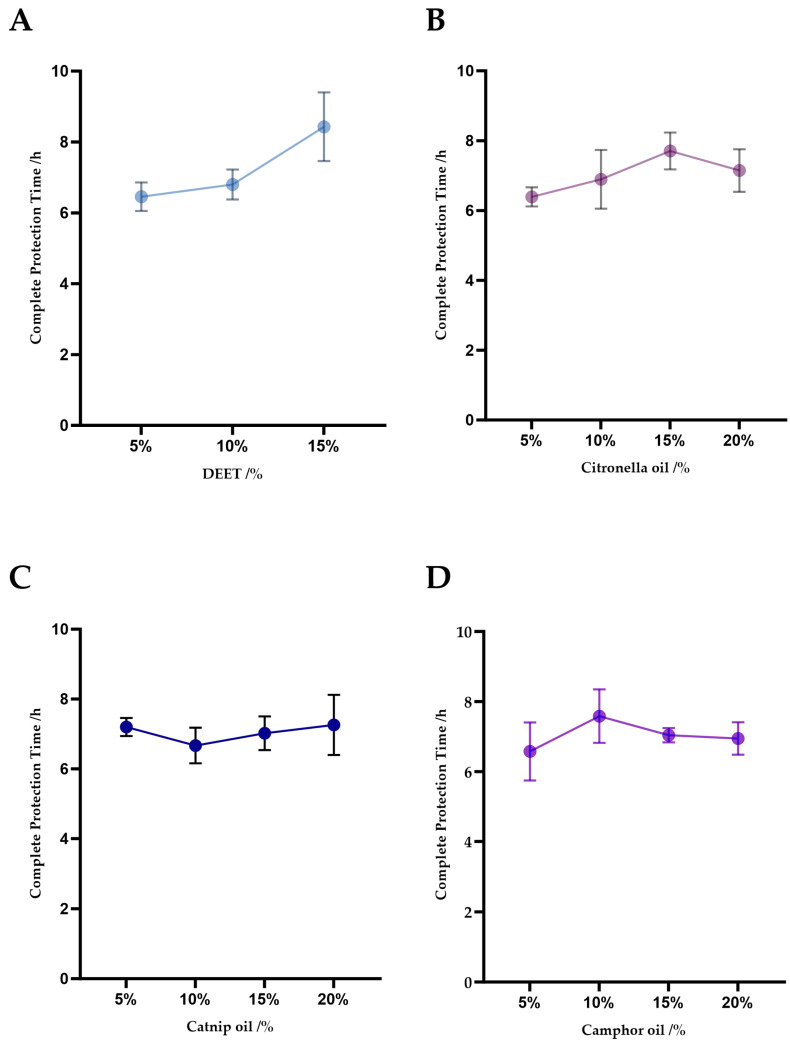
Analysis of factor-level means (*k-values*) from the orthogonal experimental design. Mean complete protection time (±*SD*, n = 6) of individual repellents at varying concentrations against *Ae. albopictus* in arm-in-cage assays. (**A**) DEET, (**B**) citronella oil, (**C**) catnip oil, (**D**) camphor oil.

**Figure 2:**
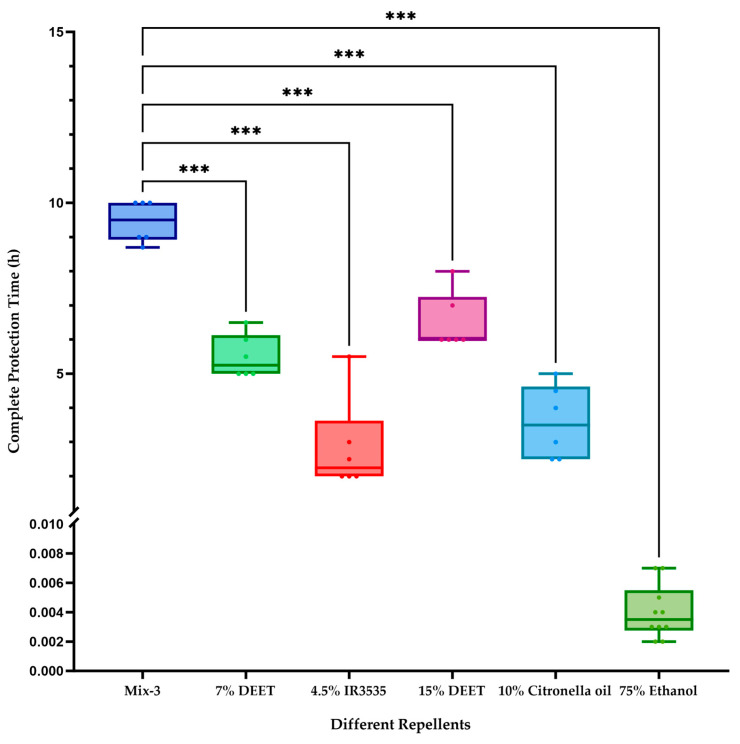
Comparison of the investigated formulation with the main components of commercial repellents. Significance: *** *p* < 0.001.

The formulation labeled as Mix-3—comprising 15% DEET, 15% citronella oil, and 10% camphor oil—yielded the longest mean protection time (Table 4, Figure 1). Individual dose–response patterns for each component are shown in Figure 1, and comparisons with commercial controls are presented in Figure 2. This optimal combination was determined through range analysis of the orthogonal experimental outcomes. The addition of catnip oil at 5–20% did not significantly improve repellent performance compared with corresponding formulations lacking catnip oil (*p* = 0.28, Table 4).

The orthogonal analysis indicated that the addition of catnip oil did not significantly improve repellent performance compared with formulations lacking it. To further validate this finding across the dataset, we classified the formulations into two distinct categories: those without catnip oil (designated as Group 1) and those containing catnip oil (designated as Group 2). Subsequently, an independent-samples *t*-test was performed to compare the mean protection times between these two groups. No statistically significant difference was detected (*t* = 0.135, *p* = 0.895) (Table 5). Formulations without catnip oil exhibited a mean protection time of 9.45 ± 0.52 h, whereas those containing catnip oil provided 9.40 ± 0.63 h of protection (Supplementary Table S2). These findings indicate that, under the experimental conditions of this study, catnip oil did not enhance repellent duration. Variance components details are provided in Supplementary Table S3.

No participants experienced skin irritation or allergic reactions throughout the experimental period, suggesting good short-term dermal tolerability of the optimized formulation [14].

### 3.2. Optimized Blend Mix-3 Emerges as a High-Performance Alternative to the Main Components of Commercial Repellents

The optimized formulation of Mix-3 (15% DEET + 15% citronella + 10% camphor) demonstrated a significantly longer duration of repellency than the primary active ingredients found in major commercial repellents. Statistical comparisons confirmed that Mix-3 exhibited significantly greater repellency than (*p* < 0.05 for all comparisons) each of the tested main components of commercial repellents (*p* < 0.05; Table 6). Specifically, Mix-3 provided an average protection time of 9.45 ± 0.52 h, outperforming 7% DEET (5.50 ± 0.55 h), 4.5% IR3535 (2.83 ± 0.55 h), 15% DEET (6.50 ± 0.55 h), and 10% citronella oil (3.58 ± 0.55 h). Notably, Mix-3 extended protection by more than 3 h relative to 15% DEET alone (Table 6, Figure 2). These results support Mix-3 as a high-performance alternative that leverages synthetic interactions among multiple agents for enhanced efficacy and to potentially improve sustainability.

**Table 6:** Multiple comparisons of the investigated formulation with the main components of commercial repellents.

Multiple Comparisons						
(I) Combination	(J) Combination	Mean Difference (I-J)	Std. Error	Sig.	95% Confidence Interval	
					Lower Bound	Upper Bound
Mix-3	7% DEET	3.95 *	0.55	0.000	2.82	5.08
	4.5% IR3535	6.62 *	0.55	0.000	5.49	7.74
	15% DEET	2.95 *	0.55	0.000	1.82	4.08
	10% citronella oil	5.87 *	0.55	0.000	4.74	6.99

* *p* < 0.05.

### 3.3. Safety Observations

Across all 96 arm-in-cage trials (16 orthogonal formulations × 6 replicates), no visible skin irritation—including erythema, edema, or pruritus—was reported or observed immediately after testing or at 24 h follow-up. These preliminary findings indicate acceptable dermal tolerability under short-term exposure conditions; however, comprehensive dermatological patch testing and long-term safety evaluations will be required prior to commercial application.

## 4. Discussion

Natural repellent systems typically comprise complex chemical mixtures of chemical constituents whose concentrations and ratios strongly influence mosquito behavioral responses [20]. In this study, the combination of DEET, citronella oil, and camphor oil yielded a synergistic formulation that substantially extended protection time compared with the individual components.

### 4.1. The Mode of Action of DEET and Essential Oils in Repelling Insects

DEET, one of the most widely used synthetic repellents, provides long-lasting protection through a multifaceted mechanism involving olfactory receptor modulation [21,22,23], disruption of gustatory neurons, and contact-mediated avoidance [24,25]. In the present study, DEET was evaluated at concentrations ranging from 5% to 20% in combination with botanical oils to investigate the efficacy of multi-component repellent formulations. Citronella oil contributes to repellent performance by human odor masking [26] and enhancing olfactory interference through activation of pathways such as *Agam*OBP4 and *Agam*ORC7 [27], thereby reducing the necessary quantity of DEET and potentially mitigating concerns associated with synthetic compounds. Camphor further prolongs repellency by eliciting aversive responses via OR49-mediated pathways [28], contributing to sustained behavioral deterrence [29,30]. Although DEET has an established safety record and is recommended, our formulation strategy intentionally reduced reliance on synthetic actives by incorporating botanical oils.

### 4.2. Implications of the Optimized Repellent Blend

The optimized blend (15% DEET, 15% citronella, 10% camphor) achieved an average protection duration of 9.45 h, outperforming formulations containing lower concentrations of DEET and botanical alternatives. This suggests that the ternary mixture may offer an effective approach for repelling *Ae. albopictus*. Although high-concentration DEET products (e.g., 33%) can provide up to 12 h of protection [31], regulatory frameworks such as the EU 2010 directive—based on animal NOEL-derived toxicity limits—recommend restricting consumer DEET concentrations to ≤15%, a level that may be insufficient for consistent protection when applications are infrequent [32,33]. Demonstrating that lower DEET concentrations can achieve high efficacy is therefore critical. Our findings provide proof-of-concept that botanical co-actives can extend protection duration when combined with moderate DEET levels.

The protection time observed for 10% citronella oil (3.87 ± 0.83 h) exceeded the values reported in recent studies. Luker et al. recorded <1 h protection against *Ae. aegypti* using contact-repellency assays [34], while a systematic review by Kongkaew et al. reported 19.7–312 min across diverse studies [35]. These discrepancies may reflect differences in mosquito species or strains, formulation matrices, application densities, and laboratory conditions. Further, semi-field trials and Phase III field evaluations following WHO PESS guidelines are recommended to validate the practical effectiveness of Mix-3 under varying environmental conditions, including perspiration, UV exposure, and airflow.

Orthogonal analysis showed that catnip oil (5–15% *w*/*w*) did not significantly enhance repellency in multi-component mixtures. Because catnip oil was not evaluated as a single-agent repellent, its intrinsic efficacy against *Ae. albopictus* remains undetermined. Prior studies indicate that catnip essential oil and its major constituent nepetalactone exhibit species-specific and dose-dependent bioactivity [4,36], but comparable data for *Ae. albopictus* using skin-exposure methods are limited. Future dose–response studies are therefore warranted. Based on its non-significant contribution in the current dataset, catnip oil was excluded from the optimized ternary formulation.

Based on published mechanistic studies, we hypothesize that the observed synergy may involve complementary sensory disruption. The combined action of synthetic and botanical components likely involves: (1) olfactory disruption, (2) odor masking, and (3) contact-induced deterrence.

### 4.3. Toxicological and Environmental Considerations

Botanical co-actives such as citronella and camphor are not inherently safer by virtue of being natural. Camphor has documented dermal absorption and case reports of hepatotoxicity [37,38], and its use during pregnancy is cautioned due to limited developmental toxicity data. Citronella oil may cause skin sensitization in susceptible individuals [39]. Our laboratory formulation employs 15% citronella for efficacy testing—this concentration exceeds regulatory limits in some jurisdictions and therefore cannot be recommended for consumer use without targeted safety evaluation and reformulation. Although no acute dermal reactions occurred in our preliminary tests, the study did not evaluate sensitization potential. Prior reports indicate that certain essential oil components may pose allergenic risks [40,41]. Comprehensive patch testing (ISO 10993-10 or OECD TG 404) [42,43] and phototoxicity evaluations are thus necessary, particularly for citronella (a known photosensitizer) and camphor. Longer-term exposure studies are also needed. Environmental toxicity is an equally important consideration. Both camphor and citronella exhibit aquatic toxicity in standard test organisms (Daphnia, fish) at relatively low concentrations [44,45]. Any progression toward commercialization should therefore include environmental fate and ecotoxicity assessments [46,47,48] alongside human safety evaluations.

Accordingly, the present work should be interpreted as a proof-of-concept efficacy assessment. Translation into consumer-ready products will require (1) sensitization and phototoxicity testing [42,49], (2) concentration adjustments to meet regulatory standards, and (3) ecological risk assessment. Further research should also examine formulation stability, user acceptability (e.g., odor, skin feel), and long-term storage characteristics. Field evaluation across diverse ecological settings and mosquito species will be essential for determining broader applicability. Collectively, the ternary formulation represents a potentially scalable approach that may support individual- and community-level vector control, although economic feasibility and environmental life-cycle analyses remain to be conducted.

## 5. Conclusions

This study developed and evaluated a hybrid botanical–synthetic repellent formulation combining 15% DEET with botanical oils, which extended protection against *Ae. albopictus* relative to 7% DEET, 15% DEET alone, and other tested formulations under controlled laboratory conditions. Validation against higher-concentration DEET formulations (≥20%) and field trials will be necessary to assess practical, real-world performance and to confirm the benefits of botanical synergists. This optimized blend (15% DEET, 15% citronella oil, and 10% camphor oil) illustrates how botanical co-actives may reduce reliance on higher DEET concentrations while maintaining effective repellency. However, comprehensive toxicological, sensitization, phototoxicity, and ecological safety assessments will be required before commercialization. These findings should therefore be regarded as proof-of-concept, demonstrating the potential utility of synergistic botanical–synthetic formulations for mosquito bite prevention.

## Figures and Tables

**Table 1:** Essential oils and synthetic repellent used in this study.

No.	Mosquito Repellents	Bioassay Standard	Manufacturer
1	DEET	GB2009 [18]	MACKLIN (Shanghai, China)
2	Citronella oil	GB2009 [18]	Yuan Ye (Shanghai, China)
3	Catnip oil	GB2009 [18]	Yuan Ye (Shanghai, China)
4	Camphor oil	GB2009 [18]	Yuan Ye (Shanghai, China)

**Table 3:** Configuration scheme derived from the orthogonal design.

Test Scheme	Factor			
	DEET (A)	Citronella Oil (B)	Catnip Oil (C)	Camphor Oil (D)
1	10%	5%	10%	10%
2	5%	15%	5%	15%
3	5%	5%	5%	5%
4	15%	20%	20%	5%
5	5%	15%	20%	10%
6	15%	15%	10%	20%
7	10%	20%	5%	20%
8	5%	10%	15%	20%
9	5%	5%	20%	20%
10	10%	15%	15%	5%
11	15%	10%	5%	10%
12	5%	10%	10%	5%
13	5%	20%	15%	10%
14	10%	10%	20%	15%
15	5%	20%	10%	15%
16	15%	5%	15%	15%

**Table 4:** Analysis of individual odor effect (*R*^2^ = 0.485, Adjusted *R*^2^ = 0.427).

Source	Type III Sum of Squares	*Df*	Mean Square	*F*-Value	*p*-Value	*η* ^2^
Corrected model	103.20	11	9.38	7.18	0.000	0.485
Intercept	4515.34	1	4515.34	3454.83	0.000	0.976
DEET	64.03	2	32.01	24.50	0.000	0.368
Citronella Oil	21.59	3	7.20	5.51	0.002	0.164
Catnip oil	5.10	3	1.70	1.30	0.28	0.044
Camphor Oil	12.50	3	4.17	3.19	0.028	0.102
Error	109.79	84	1.31			
Total	4965.41	96				
Corrected total	212.99	95				

Abbreviation: ***Df***, Degrees of Freedom.

**Table 5:** Analysis of differences in mix containing catnip oil.

	Group	N	Mean (Std. Deviation)	*t*	*p*
Result	Group 1	6	9.45 (0.61237)	0.14	0.90
	Group 2	6	9.4 (0.66633)		

## Data Availability

The original contributions presented in this study are included in the article/Supplementary Material. Further inquiries can be directed to the corresponding authors.

## Supplementary Materials

The following supporting information can be downloaded at https://www.mdpi.com/article/10.3390/insects17010098/s1, Table S1: The details of participant-level data: Age, sex, skin type (Fitzpatrick scale); Table S2: Data on differences in groups containing catmint oil; Table S3: Results of variance components analysis; Participant informed consent form: Informed consent for publication was obtained from all identifiable human participants.

## Abbreviations

The following abbreviations are used in this manuscript:

DEET	N,N-Diethyl-m-toluamide
EOs	Essential oil
ORs	Odorant receptors
*Ae. albopictus*	*Aedes albopictus*
*Ae. aegypti*	*Aedes aegypti*
EPA	Environmental Protection Agency
*Df*	Degrees of Freedom
RH	Relative humidity
TRPA1	Transient receptor potential ankyrin 1
