# Supplementary material for: Triple-Olfactory Mechanism Synergy: Development of a Long-Lasting DEET–Botanical Composite Repellent Against Aedes albopictus
Source: Insects. 2026 Jan 14;17(1):98. doi: 10.3390/insects17010098 (PMC12842024; doi:10.3390/insects17010098)
Supplement: Supplementary file 1 [file insects-17-00098-s001.zip › insects-4007796-supplementary.pdf]

Supplementary Materials Table S1:

Age	Sex	**Skin type (Fitzpatrick scale)**
35	female	type Ⅲ
35	male	type Ⅲ
21	male	type Ⅳ
21	male	type Ⅲ
22	female	type Ⅲ
20	female	type Ⅱ
21	female	type Ⅲ
23	male	type Ⅲ
22	male	type Ⅱ
20	female	type Ⅱ
19	female	type Ⅳ
21	male	type Ⅲ

Supplementary Materials TableS2. Data on Differences in Groups Containing Catmint Oil.

**Group 1**	**Group 2**
8.7	8.7
10	10
10	8.7
10	9
9	10
9	10

Supplementary Materials TableS3. Results of Variance Components Analysis

**Source**	**df**	**Mean Square**
Corrected Model	33	5.135
Intercept	1	4744.688
Formulation	15	6.827
TestOrder	7	.462
SubjectID	11	4.940
Error	62	.651
Total	96	
Corrected Total	95	

Dependent Variable: Protection time
